# Supplementary material for: Investigating the Effects of Exogenous and Endogenous 2-Arachidonoylglycerol on Retinal CB1 Cannabinoid Receptors and Reactive Microglia in Naive and Diseased Retina
Source: Int J Mol Sci. 2023 Oct 28;24(21):15689. doi: 10.3390/ijms242115689 (PMC10650178; doi:10.3390/ijms242115689)
Supplement: Supplementary file 1 [file ijms-24-15689-s001.zip › ijms-2670262-supplementary.pdf]

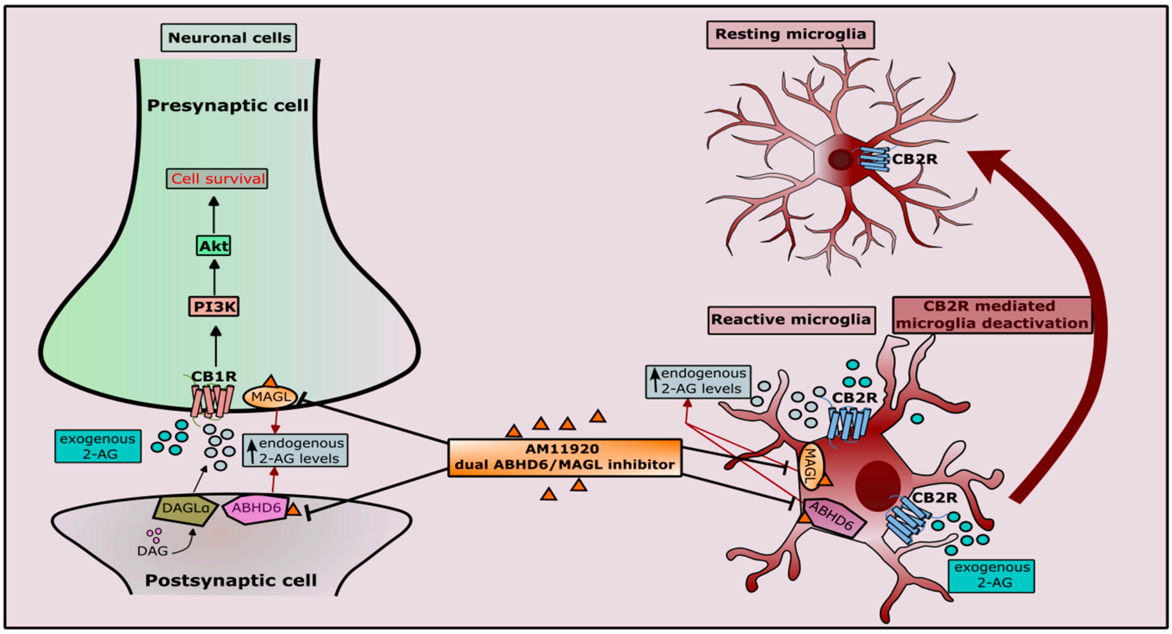

Supplementary Figure S1. Schematic representation of 2-AG's signaling pathways on retinal neurons and microglia.

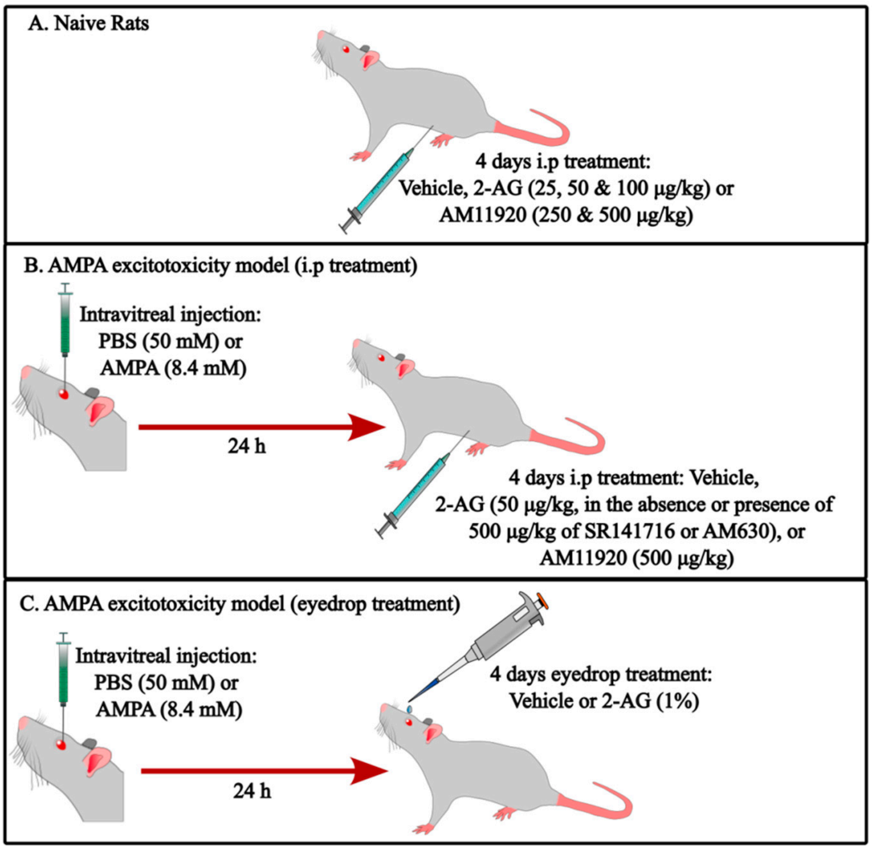

Supplementary Figure S2. Schematic representation of experimental paradigms and treatments

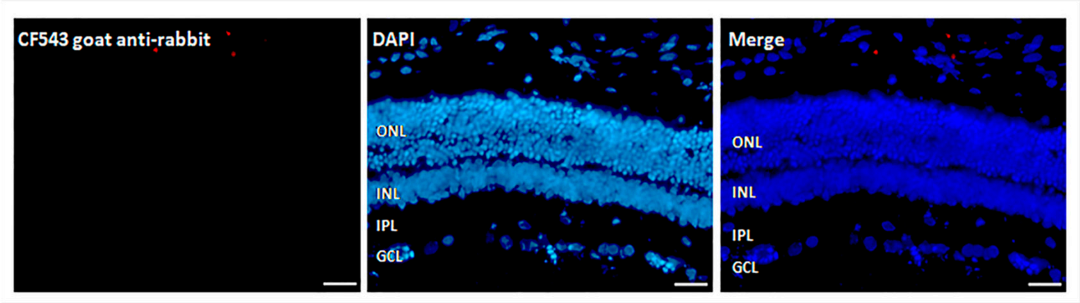

Supplementary Figure S3. Representative photomicrograph of retinal tissue stained only with the secondary antibody CF543 goat anti-rabbit (Biotium, Fremont, CA, code no. 20309, lot no. 12C0213) and DAPI nuclear staining. Magnification: x20. Scale bar: 50 µm
